# Set Configuration in Resistance Exercise: Muscle Fatigue and Cardiovascular Effects

**DOI:** 10.1371/journal.pone.0151163

**Published:** 2016-03-16

**Authors:** Dan Río-Rodríguez, Eliseo Iglesias-Soler, Miguel Fernández del Olmo

**Affiliations:** 1 Learning and Human Movement Control Group, Department of Physical Education and Sport Faculty of Sports Sciences and Physical Education, University of A Coruna, A Coruna, Spain; 2 Performance and Health Group, Department of Physical Education and Sport, Faculty of Sports Sciences and Physical Education, University of A Coruna, A Coruna, Spain; University of Debrecen, HUNGARY

## Abstract

**Purpose:**

Cardiovascular responses of traditional resistance (TS) training have been extensively explored. However, the fatigue mechanisms associated with an intra-set rest configuration (ISR) have not been investigated. This study compares two modalities of set configurations for resistance exercise that equates work to rest ratios and measures the central and peripheral fatigue in combination with cortical, hemodynamic and cardiovascular measures.

**Methods:**

11 subjects performed two isometric knee extension training sessions using TS and ISR configurations. Voluntary activation (VA), single twitch amplitude, low frequency fatigue (LFF), Mwave, motor evoked potential (MEP), short intracortical inhibition (SICI), intracortical facilitation (ICF) and heart rate variability were evaluated before and after each training session. During each session beat to beat heart rate, blood pressure and rate pressure product (RPP) were also evaluated.

**Results:**

After exercise VA decreased significantly for TS but not for ISR (P < 0.001), single twitch amplitude and LFF values were lower for TS than ISR (P < 0.004), and SICI was reduced only for the TS configuration (P = 0.049). During exercise RPP values were significantly higher for the TS than for ISR (P = 0.001). RPP correlated with VA for TS (r = -.85 P < 0.001) suggesting a relationship between central fatigue and cardiovascular stress.

**Conclusions:**

We conclude that ISR induced lower central and peripheral fatigue as well as lower cardiovascular stress in comparison with TS configuration. Our study suggests that set configuration is a key factor in the regulation of the neuromuscular and cardiovascular responses of resistance training.

## Introduction

Cardiovascular modulation and its association with resistance exercise have been the focus of extensive research[1]. The blood pressure (BP) and heart rate (HR) responses to resistance training are thought to be modulated by peripheral and central mechanisms [1,2]. The role of these mechanisms may be mediated by the configuration features (i.e. volume, intensity, number of repetitions/set, rests between sets) of the resistance training, although this relationship has not been studied extensively. Traditional set configuration (TS) is the most common procedure of resistance training and consists of performing each repetition of a set without rest until failure [3]. This configuration induces fatigue and discomfort [4]. In addition, BP and HR rises proportionally with successive repetitions within a set [2], and the rate at which BP increases is related with both the intensity and the length of a set [5].

A novel approach to the set configuration is the intra-set rest configuration (ISR), which introduces pauses between single or small groups of repetitions (i.e cluster training). Thus, the perceived effort is reduced while the performance is maintained [4]. This type of intermittent resistance training reduces the BP response during dynamic [6] and isometric exercise [7] in comparison with TS configuration. The differential modulation of the BP in response to TS and ISR may be due to the differences in the work-to-rest ratios (i.e. effort vs. resting time) between the configurations. However, a previous study has shown that the lower BP response during ISR in comparison with TS remained even when the work-to-rest ratios are equated for both configurations [8], suggesting that other factors underlie these differences. We proposed that ISR induces lower levels of muscle fatigue, compared with TS, since this configuration is associated with improved mechanical performance [9] and less perceived effort [4,8,10]. Moreover, peripheral and central fatigue may play differential roles in each configuration. Peripheral fatigue reflects an impairment at, or distal to the neuromuscular junction, and can be evaluated by recording the twitch force that is induced by peripheral nerve stimulation while the muscle is at rest [11]. Central fatigue indicates a failure to drive the motor neurons adequately [12], and can be tested by recording the force evoked by nerve stimulation during a maximal voluntary effort [13]. In addition, muscle fatigue can be associated with changes that are elicited by transcranial magnetic stimulation (TMS), in the motor evoked potential (MEP) [14], short intracortical inhibition (SICI) [14] and intracortical facilitation (ICF) [15,16], although these changes may be difficult to interpret [17].

There are no studies to date that have investigated central and peripheral fatigue induced by TS and ISR configurations, with an equal work-to-rest ratio, and their relationship with cardiovascular changes. Thus, the aim of this study was to investigate differences in the neurophysiological, mechanical and cardiovascular acute responses of TS and ISR configurations matched for volume, intensity and work-to-rest ratio. This will allow us to test the hypothesis that the levels of muscle fatigue induced by different resistance exercise configurations account for the differences in their cardiovascular response. This will provide further insight into the mechanisms underlying the cardiovascular modulation in response to changes in the resistance set configuration.

## Materials and Methods

### Subjects

Eleven young males participated in this study (age 21.0 ± 2, height 177.2 ± 0.08 cm, weight 72.4 ± 6.6 kg). The subjects were recruited from the Institute of Physical Education and Sport of A Coruña, Spain. All the subjects were physically active and none of them reported neurological impairment, lower limb injuries and/or contraindications to TMS. Written informed consent was obtained from all the subjects after a full explanation of the procedures and risks involved All the experimental procedures were approved by University of A Coruña ethics committee and conformed to the Declaration of Helsinki.

### Experimental Procedure

**Fig 1:**
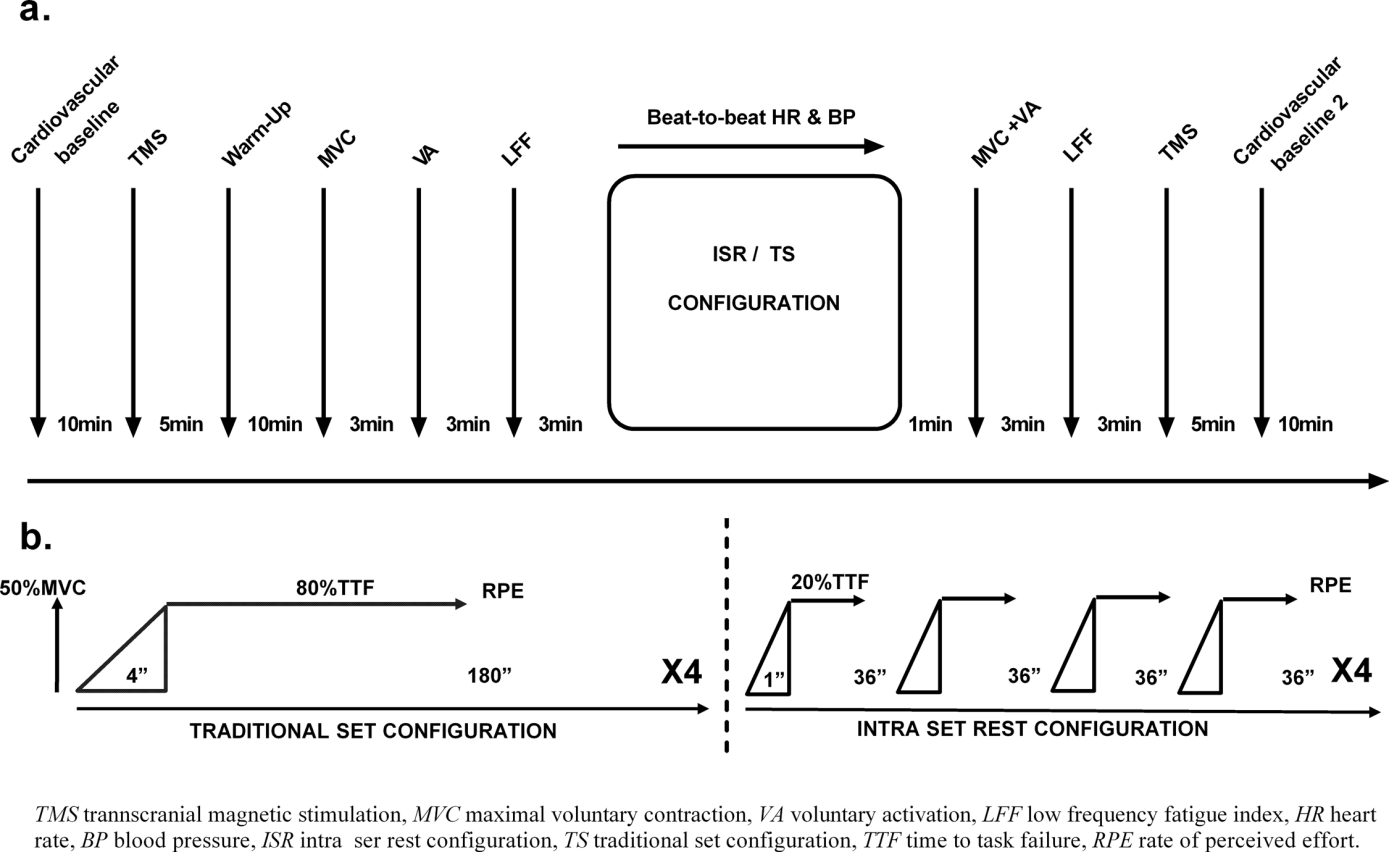
Schematic representation of the experimental protocol. (a) time line of measurements. (b) the training protocols performed in each session,. Each protocol (TS/ISR) utilized the same intensity, total muscle contraction and rest time.

Each subject participated in five familiarization and three experimental sessions. The protocol is described in Fig 1A. The familiarization sessions were used to familiarize the subjects with the maximal voluntary contractions, electrical and magnetic stimulations and with the rate of perceived effort scale. The first experimental session was conducted to calculate the maximal voluntary isometric contraction (MVC) during a knee extension exercise and the time to task failure (TTF) at a 50% of MVC. The other two experimental sessions correspond to two resistance-training sessions: with a traditional set configuration (TS) or with intra-set rest configuration (ISR). The order of TS and ISR sessions were counterbalanced, separated by 1 week and conducted at the same time of day for each subject. Each training session started with hemodynamic recordings and cortical measurements (using transcranial magnetic stimulation of the motor cortex) with the subject at rest. Then, after a standardized warm-up the subjects performed dynamic and neuromuscular assessments (maximal voluntary contractions and electrical stimulation, respectively). The subjects then started the exercising procedure (with TS or ISR configuration) during which cardiovascular parameters were continuously recorded. Immediately after the exercise, dynamic and neuromuscular assessments were recorded, and the rate of perceived effort was reported by the subjects. Ten minutes later, cortical and cardiovascular recordings were obtained.

### Maximal Voluntary Contraction

Subjects were seated in a modified knee extension machine (BF100, Biotech Bioiso, Brazil) attached to a force cell (sensitivity: 2 mV/V and 0.0028 V/N; NL63-200, Digitimer Ltd, Welwyn Garden City, UK) with the hips flexed at 90° and the right knee flexed at 90° and firmly strapped to the lever arm of the machine. To ensure that participants only used the knee extensors special care was taken positioning the lever arm above the ankle, and a belt was used to avoid hip and trunk movement. Participants were asked to perform an MVC “as fast and as forcefully as possible” and to maintain it for 4 seconds [18]. We followed the recommendations of Gandevia [12] for a reliable MVC measurement: (i) feedback of performance was given during all the voluntary contractions (visual display), (ii) appropriate standardized verbal encouragement was given by the investigators, (iii) subjects were allowed to reject efforts that they did not regard as ‘‘maximal’ and attempted another trial 3 minutes later.

### EMG Recordings

Electromyographic (EMG) signals were recorded using bipolar self-adhesive Ag/AgCl electrodes of 10-mm diameter (F9079P, FIAB, Vicchio, Italy) in bipolar configuration of the rectus femoris (RF) and biceps femoris following the SENIAM recommendations [19], with an inter-electrode distance of 25 mm and with the reference electrode on the patella. The position of the electrodes was marked on the skin so that these were used in the subsequent session. The recording sites were shaved, abraded and cleaned with isopropyl alcohol to obtain low impedance (Z, 5kΩ). EMG signals were amplified and filtered with a bandwidth frequency ranging from 10 Hz to 1 kHz (gain = 1,000). The EMG signals were simultaneously digitized with the torque signals, using an acquisition card at a sampling rate of 5 kHz per channel (Digitimer D360, Welwyn Garden City, UK) and stored for later analysis on a computer with a custom built Signal script.

Force and EMG signals were synchronized using a Power 1401 A-D converter and Signal software [Cambridge Electronics Design (CED), Cambridge, UK].

### Electrical Stimulation

*M wave*. Electrical stimulation was used to activate the femoral nerve. A ball probe cathode was manually pressed over the femoral triangle 3-5cm below the inguinal ligament. The anode, a 130×80mm self-adhesive electrode (Cefar-Compex Scandinavia AB, Sweden), was applied to the gluteal fold. Square-wave pulses with a width of 1 ms at a maximal voltage of 400 V from a constant current stimulator (Digitimer DS7A, Welwyn Garden City, UK) were delivered to the resting muscle. The optimal stimulation intensity for a single stimulus was determined by increasing the intensity until the amplitude of the evoked twitch showed no further increase (M_max_). The intensity used for subsequent stimulation techniques was 120% of that which evoked a maximal twitch torque with subsequent M_max_ of the RF (140–240 mA).

#### Low Frequency Fatigue

Two electrical stimuli at 100-Hz (Db100) and 10-Hz (Db10) were delivered 4 s apart over the femoral nerve two seconds after an MVC of the knee extensors [20].

#### Twitch interpolation

Twitch interpolation technique [13] was applied to the knee extensors. During an MVC, a superimposing supramaximal electrical stimulus was delivered to the femoral nerve, followed by a second electrical stimulus 1.5 s after the end of the MVC.

### Transcranial Magnetic Stimulation (TMS) of the Motor Cortex

Single TMS pulses of 1-ms duration Magstim BiStim 200^2^, The Magstim Company, Dyfed, UK) were delivered via a concave double-cone coil (diameter: 110 mm; maximum output: 1.4 T). The handle of the TMS coil was positioned over the vertex of the head and held tangential to the skull in an anterior–posterior orientation. The coil was positioned over the left motor cortex and the orientation of the coil was determined by localizing the largest motor evoked potential (MEP) in the right RF, with the lowest motor response in the biceps femoris. The optimal stimulation site was marked with an indelible red marker to ensure reproducibility of the stimulus conditions for each subject throughout the sessions. The resting motor threshold (RMT) was determined as the minimum stimulus intensity required to elicit an MEP in the RF of at least 50 μV in 3 of 5 consecutive trials.

Short interval intracortical inhibition (SICI) and intracortical facilitation (ICF) were recorded using techniques which have been previously described [21,22]. Paired magnetic stimuli at different interstimulus intervals were applied at the optimal scalp site for evoking responses in the right RF while the subject was at rest. The test (second) stimulus was set to intensity sufficient to evoke a response in the RF of approximately 0.5–1 mV. The conditioning (first) stimulus was at intensity 80% of stimulator output below the resting motor threshold for the target muscle. The interval between conditioning and test stimuli was 3 ms for the investigation of SICI and 15 ms for the investigation of ICF. Inhibitory, excitatory timings and TMS alone were incorporated into a single block of 45 stimuli. Therefore, in total there were 15 trials for each condition, and the orders of presentation of the conditions were randomized.

### Cardiovascular Recordings

Cardiovascular and autonomic parameters were recorded using a non-invasive measurement system Task Force Monitor (CNSystems Medizintechnik GmbH–Austria), with 4 electrodes for electrocardiograph recording and two pneumatic plethysmography devices placed on the first phalange of the second and third fingers of the left hand. The distal pressure measurement was regularly corrected by an oscillometric measurement taken from the right brachial artery. Recordings were conducted at a sample rate of 1000 Hz.

### Rate of Perceived Effort (RPE)

Rate of perceived effort was recorded using a visual scale with verbal anchors (OMNI-Scale) [23]. Before each exercise protocol the standard definition of the perceived exertion and instructions for the mode specific OMNI Scale were read to subjects. The scale was placed in front of the subjects below the feedback screen during the exercise trial.

### Experimental Sessions

#### First experimental session

The MVC during an isometric knee extension exercise and TTF at a 50% of MVC were recorded for each subject. The MVC was performed according with the instructions described previously. Five minutes later, the TTF test was performed. The subjects were required to exert continuously a force of 50% MVC as long as they could. They were encouraged during the duration of the test. The test was considered to be completed when the subjects were no longer able to achieve the required force.

#### Training sessions

Each training session began with a standardized warm-up that included 5 min of cycling on a cycle ergometer (Monark 828E; Monark Exercise AB, Vansbro, Sweden) at a power output equivalent to 60 W followed by 5 submaximal isometric knee contractions (2x50-2x70-1x90% MVC). After this warm-up the subjects perform one of the training sessions.

The TS configuration session consisted of 4 sets of 50% MVC isometric knee extensors. The duration of each set was adjusted to 80% of the TTF for each subject (obtained in the first experimental session). The rest interval between sets was 180 seconds.

The ISR configuration session consisted of 16 sets of 50% MVC isometric knee extensors. The duration of each set was adjusted to 20% of the TTF for each subject. The rest interval between sets was 36 seconds.

The total time of muscle contraction and at rest for each subject was equivalent between training sessions (i.e. a subject with a TTF of 60 seconds performed both training sessions with a total muscle contraction time of 192 seconds and a total rest time of 540 seconds). In order to equate the muscle contraction time, each contraction started with a progressive slope of 4 seconds in TS and 1 second in ISR. Fig 1B represents graphically both training set configurations.

### Data Analysis

#### Electrical stimulation

The amplitude of the M_max_ evoked during a single supramaximal electrical stimulus was recorded. Maximal rates of force development (ST-RFD) and half relaxation time (ST-½RRT) of the single twitch were measured using femoral nerve stimulation. Rate of force development was calculated as the maximum value of the first derivative over time of the force-time curve during a single twitch. Rate of relaxation was calculated from peak torque to half peak torque

Maximal voluntary activation was quantified using the twitch interpolation technique [13]. Briefly, the force produced by a superimposed twitch delivered during the MVC was compared with the force produced by a single twitch delivered during relaxation ∼2 s after the MVC:

Voluntary activation (%) = [1 –(superimposed twich/resting twicth)]×100.

Low frequency fatigue index (LFF) was quantified as the ratio between the torque produced with 10 Hz stimulation and that produced with 100 Hz (10 Hz / 100 Hz).

#### TMS

The peak-to-peak amplitude of the MEP was measured offline. The MEP measured on the RF was normalized to Mmax [17]. SICI and ICF values were expressed as a percentage of the unconditioned test MEP amplitude.

#### Cardiovascular variables

Mean systolic blood pressure (SBP_mean_), diastolic blood pressure (DBP_mean_), mean arterial pressure (MAP_mean_) and heart rate (HR_mean_) were recorded beat to beat and averaged at the end of each training session including the pauses. Rate-pressure Product (RPP) were calculated as the heart rate multiplied by the MAP (HR x MAP) as described in [24]. Rate-pressure product is an indicator of the cardiac workload and the oxygen requirement of the heart.

Heart rate variability indices were analyzed according to the Task Force of the European Society of Cardiology and North American Society of Pacing and Electrophysiology [25] and were calculated for the last 5 minutes of a 10 minute window before and after the exercise. Mean R-R intervals (RRI) power densities in the low (LF, 0.04–0.15 Hz) and high frequency band (HF, > 0.15–0.40 Hz) were analyzed with autoregressive frequency domain methods [26]. Occasional ectopic beats and artefacts were visually identified and replaced with interpolated RRI [27].

The baroreflex sensitivity (BRS) was determined using the sequence method as previously described [28]. The time series for RRI and SBP were scanned for sequences in which both RRI and SBP concurrently increased (BRS-Up) or decreased (BRS-Down) for a minimum of three consecutive beats. Task Force Software sets the threshold values to 1 mmHG in SPB and 6 ms in RRI [29]. BRS was computed as the ratio RRI/SBP (ms·mmHg^-1^) during baseline rest and the end of the exercise.

#### Rate of perceived effort (RPE)

A general perception of effort (RPE-Overall) and local perceived effort (RPE-Leg) were reported by the subjects at the end of each set using the OMNI Scale [23,30]. The values reported by the subjects were averaged for each set configuration in order to obtain a mean RPE for each resistance exercise configuration.

### Statistical Analysis

Analyses of variances (ANOVA) of repeated measurements with time (before, during and after the exercise) and set configuration (TS, ISR) as main factors were applied to the mean and the maximum values of the cardiovascular variables (HR, SBP, DBP, MAP, RPP).

ANOVAs of repeated measurements with time(before vs. after the exercise) and set configuration (TS vs. ISR) as main factor were performed for the following variables: MEP, SICI, ICF amplitude, Total Power, LF, HF, BRS-Up, BRS-Down, MVC, M_max_, VA, ST, ST-RFD, ST-½RRT, LFF. Post-hoc analyses were conducted using Bonferroni correction.

Normal distribution was checked by using a Shapiro-Wilk test of normality. When normality could not be assumed, logarithmic transforms were completed as required. Student’s paired t test was used to compare RPE between set configurations. Correlations between variables were determined using Pearson product moment or Spearman rho coefficients as appropriate in order to explore associations between cardiovascular, mechanical and neurophysiological variables. Statistical analysis was conducted with SPSS software version 15.0 (SPSS, Chicago, IL). Statistical significance was set at P≤ 0.05.

## Results

All the subjects completed all the familiarization and experimental sessions. They were able to perform the training sessions and the tests as required. One subject was excluded from the analysis of cortical parameters since no MEP could be induced by TMS stimulation. The analyses for the remaining parameters were performed with and without this subject and no differences in the results were found. Therefore, this subject was only excluded from the MEP analysis. No significant differences were found in the baselines values between set configurations for all the variables recorded (P-values ranged from 0.188 to 0.854).

### Maximal Voluntary Contractions

**Fig 2:**
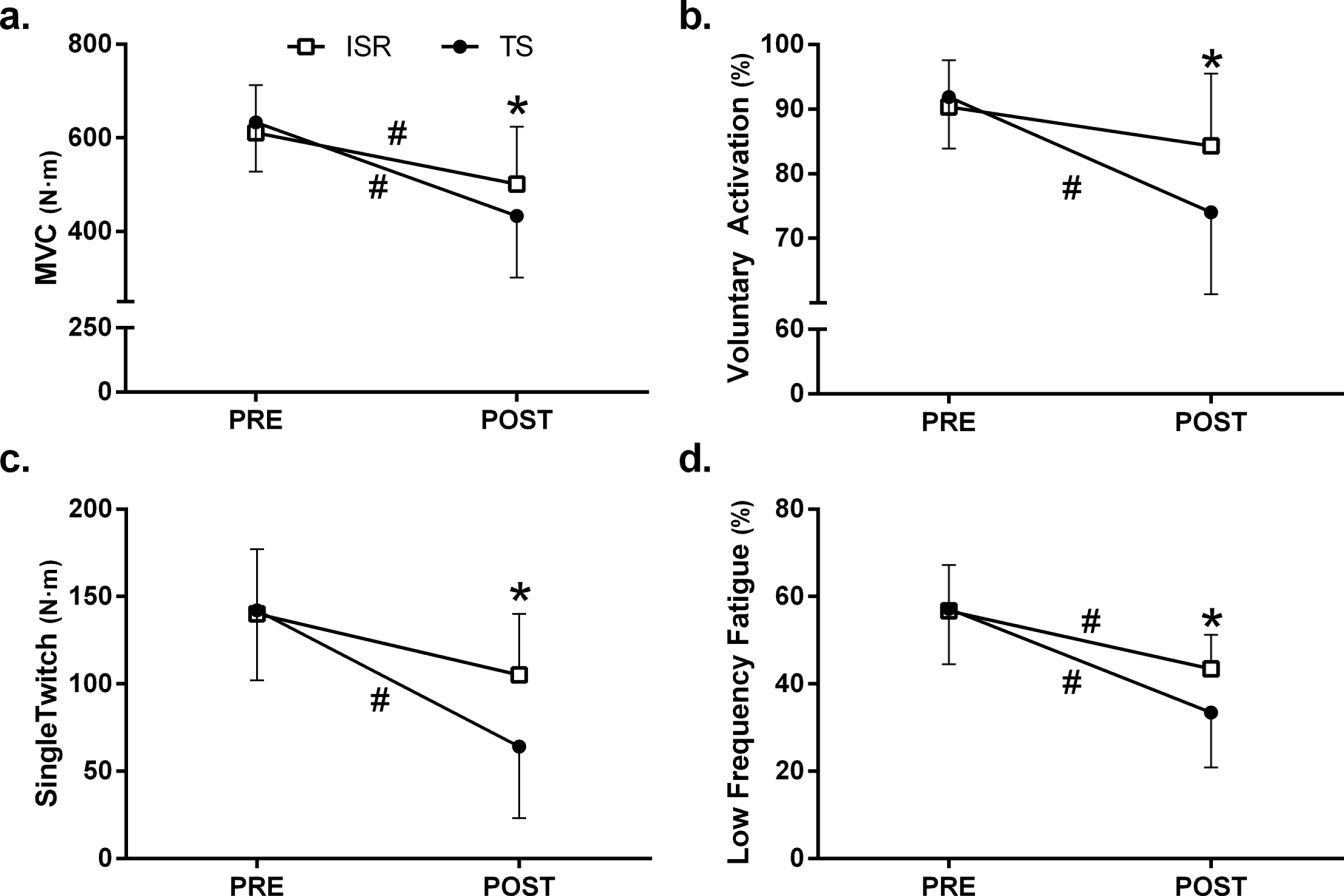
Changes in maximal voluntary contraction (a), voluntary activation (b), single twitch (c), low frequency fatigue (d) after the performance of traditional set (black circles) and intra set rest configuration (white squares). Mean and SD values are displayed. *Significant differences between set configurations. #Significant differences between with PRE and POST values.

The results of the MVC are displayed in Fig 2A. There was a significant decrease in the MVC in both set configurations (P ≤ 0.001). The MVC post-exercise was significantly lower for the TS in comparison with the ISR configuration (P = 0.019) indicating a greater decrease in MVC for TS compared with ISR (see Table 1).

**Table 1:** Mean, SD and P values of the mechanical effects, central and peripheral fatigue of each set configuration.

		ISR	TS		ANOVA	
VARIABLE (unit)	MOMENT	MEAN	MEAN	SET	MOMENT	INTERACTION
**MVC (N·m)**	PRE	611 (±102)	633 (±105)	0,184	<0,001	0,003
** **	POST	501 (±123) #	433 (±132) # *			
**VA (%)**	PRE	90 (±7)	92 (±8)	0,153	<0,001	0,026
** **	POST	84 (±11)	74 (±13) # *			
**LFF (A.U.)**	PRE	0,57 (±0,08)	0,57 (±013)	0,029	<0,001	0,015
** **	POST	0,43 (±011) #	0,33 (±0,13) # *			
**Db100Hz (N·m)**	PRE	260 (±46)	267 (±36)	0,055	<0,001	0,003
** **	POST	254 (±48)	200 (±59) # *			
**Db10Hz (N·m)**	PRE	147 (±38)	151 (±37)	0,042	<0,001	0,004
** **	POST	111 (±35) #	71 (±42) # *			
**Single Twitch (N·m)**	PRE	140 (±37)	142 (±40)	0,001	<0,001	<0,001
** **	POST	105 (±36)	64 (±41) # *			
**ST-RFD (N·m·s**^**-1**^**)**	PRE	2539 (±903)	2680 (±764)	0,004	0,619	<0,001
** **	POST	3330(±848) #	1642 (±992) # *			
**ST-½RRT (N·m·s**^**-1**^**)**	PRE	-1524(±504)	-1531 (±589)	0,002	0,191	0,001
** **	POST	-2344 (±742) #	-1114 (±848) # *			

*MVC* maximal voluntary contraction, *VA* voluntary activation, *LFF* low frequency fatigue index *Db100Hz* double electrical pulse at 100Hz, *Db10Hz* double electrical pulse at 10Hz, *Single Twitch* single electrical pulse, *ST-RFD* Rate of force development of single twitch, *ST-½RRT* Rate of relaxation of single twitch, *ISR* intra set rest configuration, *TS* traditional set configuration.

* Significant differences between set configurations.

# Significant differences compared with PRE values.

### Single Twitch

Both set configurations lead to a reduction of the Single Twitch Torque (Table 1). However, this reduction was significant in the TS configuration (P < 0.001), while the ISR configuration only showed a tendency for a reduction (P = 0.053). In addition, the post-exercise Single Twitch Torque values were significantly lower in the traditional in comparison with the ISR configuration (P < 0.001) (Fig 2C). The analysis for ST-RFD revealed that while the traditional set configuration induced a significant decrease for the ST-RFD (P = 0.008), the ISR configuration lead to an increase of this parameter (P = 0.016). Finally, ST- ½RRT increased significantly only in the ISR configuration (P = 0.001) and these values post-exercise were significantly higher for ISR in comparison with TS configuration (P < 0.001).

**Fig 3:**
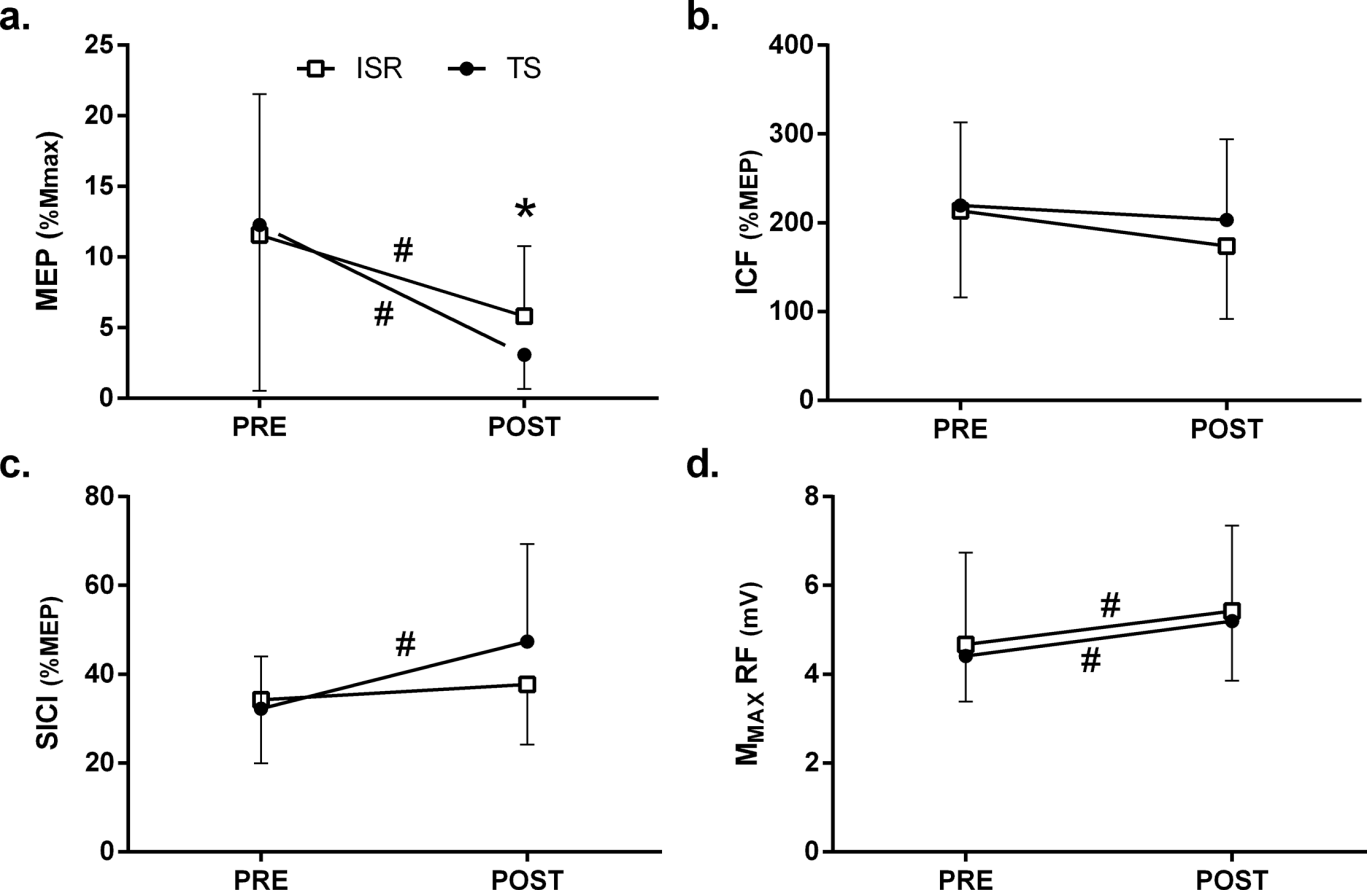
Changes in motor evoked potentials (a), intracortical facilitation (b), short intracortical inhibition (c) maximal wave of rectus femoris (d) after the performance of traditional set (black circles) and intra set rest configuration (white squares). Mean and SD values are displayed. *Significant differences between set configurations. #Significant differences between PRE and post values.

The analysis of the M-Wave for the Rectus Femoris (Fig 3D) showed a significant effect for the time (F_1,10_ = 17.091, P = 0.002) but no main effect for set configuration (F_1,10_ = 0.234, P = 0.639), nor a set configuration x time interaction (F_1,10_ = 0.020, P = 0.890) (Fig 2D).

### Low Frequency Fatigue

The analysis of the LFF revealed that LFF ratio decreased significantly after each exercise configuration (P < 0.001 for TS and ISR configurations) (Table 1). In addition, LFF ratio post-exercise was significantly lower in the traditional compared with the ISR configuration (P = 0.003) (Fig 2D). While similar results were found for the Db10Hz component of the LFF, this was not the case for the high frequency component measured with Db100Hz. The Db10Hz decreased significantly for both set configurations (P < 0.001) and the values post-exercise were significantly lower in TS compared with ISR (P = 0.003). However, Db100Hz values decreased significantly only in the TS configuration (P = 0.001) and these post-exercise values were significantly lower for the TS in comparison with the ISR configuration (P = 0.007).

### Voluntary Activation

The analysis of the Voluntary Activation showed a significant reduction of this parameter in the traditional (P < 0.001) but not in the ISR configuration (Fig 2B and Table 1).

### Transcranial Magnetic Stimulation

The analysis of the MEP amplitude normalized with the M-wave (Fig 3A) showed a decrease in MEP amplitude after exercise for both set configurations (50% and 75% reduction for ISR and TS, respectively). This reduction was only significantly for the TS configuration (P = 0.031) although a tendency to significance was found for the ISR configuration (P = 0.053). The differences in the MEP amplitudes post-exercise between set configurations were statistically significant (P = 0.034). The analysis of SICI values revealed higher post-exercise values for the TS configuration compared with baseline (ΔTS = 46%, P = 0.049) and ISR (ΔISR = 8%, P = 0.516), indicating less intracortical inhibition for the latter although no statistical differences were found between groups after exercise (P = 0.109). There were no significant differences in the ICF values between or after the exercise set configurations (Fig 3B).

### Cardiovascular Response during Exercise

The analysis for the HR_mean_ showed that the baseline values of both set configurations had a significant increase during exercise (P ≤ 0.003) returning to baseline values in ISR but not in TS after exercise (P = 0.003). Traditional set configuration lead to higher HR values during and after exercise compared with ISR (P < 0.001 and P = 0.001) (see Table 2). The HR_max_ remains elevated during and after exercise (P ≤ 0.001 and P = 0.013, respectively). Post-hoc analysis showed higher values for TS during (P ≤ 0.001) and after exercise (P = 0.001) with respect to ISR configuration.

**Table 2:** Mean, SD and P values of the mean cardiovascular response before, during and after exercise

		ISR	TS		ANOVA	
VARIABLE (units)	MOMENT	MEAN (±SD)	MEAN (±SD)	SET	MOMENT	INTERACTION
**HR**_**mean**_ **(bpm)**	PRE	66(±14)	66(±13)	<0,001	<0,001	<0,001
** **	DUR	88 (±15) # ¥	108(±14) # ¥ *			
** **	POST	66(±13)	75(±12) # *			
**MAP**_**mean**_ **(mmHg)**	PRE	85(±6)	90(±5)	0,084	<0,001	0,283
** **	DUR	103(±14)	116(±16)			
** **	POST	90(±8)	90(±17)			
**SBP**_**mean**_ **(mmHg)**	PRE	113(±7)	118(±8)	0,114	0,007	0,358
** **	DUR	130(±21)	144(±21)			
** **	POST	121(±6)	120(±21)			
**DBP**_**mean**_ **(mmHg)**	PRE	67(±7)	72(±5)	0,136	<0,001	0,305
** **	DUR	84(±12)	98(±14)			
** **	POST	70(±10)	72(±18)			
**RPP (mmHg·bpm)**	PRE	5629(±1411)	5928(±1233)	<0,001	<0,001	0,01
** **	DUR	9007(±1739) #	12425(±1841) # ¥ *			
** **	POST	5957(±1155)	6835(±1827)			

*HR*_*mean*_ mean heart rate, *MAP*_*mean*_ mean value of mean arterial pressure, *SBP*_*mean*_ mean systolic blood pressure, *DBP*_*mean*_ mean diastolic blood pressure, *RPP* rate pressure product, *ISR* intra set rest configuration, *TS* traditional set configuration.

* Significant differences between set configurations.

# Significant differences compared to PRE values.

¥ Significant differences compared to POST values

For the MAP_mean_, the analysis revealed higher values for MAP during exercise compared with baseline values and after exercise (P < 0.001 and P = 0.003, respectively) and no differences before and after exercise (P > 0.05). The MAP_max_ analysis showed higher values for TS during exercise (P = 0.001) compared with ISR.

The analysis for the SBP_mean_ revealed higher SBP values for SBP during exercise compared with values at baseline and after exercise (P = 0.006 and P = 0.005, respectively) and no differences before and after exercise (P > 0.05). The SBP_max_ showed higher values for TS during exercise (P = 0.003) compared with ISR.

The DBP_mean_ analysis showed higher DBP values during exercise compared with values at baseline and after exercise (P ≤ 0.001 and P = 0.002 respectively) and no differences before and after exercise (P > 0.05). In addition, TS induced higher values compared with ISR (P = 0.048). The DBP_max_ showed higher values for TS during exercise (P = 0.001) compared with ISR.

The rate-pressure product increased during exercise compared with baseline (P < 0.001) with significant differences between ISR and TS (P < 0.001). Significantly higher values were observed for TS compared with ISR during exercise (P = 0.001) and returning to baseline values after exercise (P = 0.063).

The HF spectral component of the heart rate variability decreased significantly only in the traditional set configuration (P = 0.013) and these post-exercise values were lower for the TS in comparison with the ISR configuration (P = 0.023) (see Table 3). The analysis for LF revealed that TS post-exercise values had a tendency to decrease (P = 0.051). Post exercise values for TS were significantly lower compared with ISR (P = 0.012).

**Table 3:** Mean, SD and P value of heart rate variability and baroreflex sensitivity before and after exercise.

		ISR	TS		ANOVA	
VARIABLE (unit)	MOMENT	MEAN	MEAN	SET	MOMENT	INTERACTION
**ln-HF (ms**^**2**^**)**	PRE	6,48 (±1,11)	6,62(±1,13)	0,221	0,268	0,005
	POST	6,79(±1,21)	5,87(±0,95) # *			
**ln-LF (ms**^**2**^**)**	PRE	6,91(±0,64)	7,06(±0,90)	0,258	0,702	0,015
	POST	7,28(±0,89)	6,80(±0,71) *			
**ln-TP (ms**^**2**^**)**	PRE	7,73(±0,76)	8,05(±0,98)	0,304	0,519	<0,001
	POST	8,11(±0,86)	7,42(±0,71) # *			
**ln-BRS-Up (ms.mmHg)**	PRE	3,28 (±0,63)	3,00 (±0,52)	0,06	0,013	0,007
	POST	3,23(±0,70)	2,58(±0,46) # *			
**ln-BRS-Down (ms.mmHg)**	PRE	3,03 (±0,63)	2,83 (±0,55)	0,193	0,003	0,022
** **	POST	3,12(±0,41)	2,34(±0,43) # *			

*Ln-HF* Natural logarithm of high frequency band, *Ln-LF* Natural logarithm of low frequency band, *Ln-TP* Natural logarithm of total spectrum power, *Ln-BRS-Up* Natural logarithm of ascending sequences of baroreflex sensitivity, *Ln-BRS-Down* Natural logarithm of descending sequences of baroreflex sensitivity *ISR* intra set rest configuration, *TS* traditional set configuration.

* Significant differences between set configurations.

# Significant differences compared with PRE values.

The Total Power for TS showed a significant reduction after exercise (P = 0.019) while ISR showed no significant differences (P = 0.066). TS values were lower compared with ISR values (P = 0.002).

Baroreflex activation (BRS-Up) analysis revealed a significant decrease for the TS (P = 0.002) but not for the ISR configuration. The BRS-Up after the TS configuration was significantly higher compared with ISR (P = 0.002). The baroreflex inhibition (BRS-Down) post exercise was significantly lower for the traditional set configuration (P ≤ 0.001). Differences between set configurations were found (P = 0.020) indicating an impaired baroreflex sensitivity for TS configuration.

### Perception of Effort

The comparison of RPE-Overall between set configurations revealed significant differences (t = -4.1, P = 0.002). When the subjects were asked to distinguish peripheral sensations (RPE-Leg), significant differences were found (t = -4.378, P = 0.001) between set configurations.

### Correlations

The increase in Rate-Pressure Product (RPP), from pre to during exercise values, correlated significantly and negatively with the change in the Voluntary Activation post-exercise for the TS configuration session (r = -0.85, P < 0.001). The increase in the MAP, from pre to during exercise values, showed a significant and negative correlation with the change in VA for the TS configuration session (r = -0.65, P = 0.03). In addition, the decrease in the SICI values correlated significantly and negatively with the change in the BRS-Down for the TS configuration (r = -0.71, P = 0.014). There were no correlations between these variables for the ISR configuration session.

The value of RPE-Leg in traditional training had a strong negative correlation with the change in the Low Frequency Fatigue index (r = -0.75, P = 0.008). The RPE-Leg also showed a strong negative relationship with the change of low frequency doublet pulse, Db10 Hz (r = -0.75, P = 0.008). Finally, the change in the high frequency electrical evoked pulse (Db100 Hz) showed a significant positive correlation with the LF spectral component of HRV (r = 0.65, P = 0.030).

## Discussion

The aim of this study was to explore and compare the neurophysiological, mechanical and hemodynamic acute responses of traditional and intra-set rest configurations with equal volume, intensity and work-to-rest ratios. Our main findings show that ISR configuration is associated with lower levels of central and peripheral fatigue, as well as lower hemodynamic and cardiovascular stress, in comparison with a traditional set configuration. Interestingly, central fatigue was associated with cardiovascular stress during the traditional resistance exercise configuration but not during the intra-set rest configuration, suggesting that central fatigue processes are linked with the intensity of the cardiovascular responses. Therefore, the current findings suggest that the set configuration has a key role in determining the levels of fatigue and cardiovascular effects that are induced by resistance exercises.

### Set Configuration Effects on Muscle Function

The intra-set rest configuration resulted in a loss of MVC of 18%, whereas TS configuration induced a greater decrement (32%). Previous studies have shown that ISR is associated with a superior mechanical performance compared with that of TS configuration [8,9]. Our results expand on these findings and show this for isometric exercises as well.

The traditional set but not the ISR configuration lead to peripheral fatigue, since the rate of force development and the rate of relaxation time of the evoked torque twitches were impaired after the TS configuration whereas the ISR lead to an improvement of these variables. These opposite responses to a single twitch may be explained by the coexistence of fatigue and potentiation mechanisms that occur immediately after exercise [11,12,17,31]. The M-wave amplitude increases that were observed after both set configurations suggest that there is a post-activation potentiation effect. However, our findings suggest that only the ISR, and not the TS configuration, benefited from this potentiation effect. It is plausible that the TS configuration lead to a reduction in Na^+^-K^+^ ATPase activity [32] and impair sarcoplasmic reticulum Ca^2+^ release and uptake [33], as a result of the longer relative contraction times in comparison with the ISR configuration. Although, the total muscle contraction time was identical for both configurations, the TS was characterized by 4 periods of long contractions in comparison with the 16 periods of short contractions in the ISR configuration. Longer muscle contraction periods may have a greater impact on the metabolic profile of the excitation-contraction coupling [34]. Furthermore, both set configurations induced low frequency fatigue, although this fatigue was more intense after TS than after ISR. However, the nature of these changes was different for each configuration. While the TS configuration induced lower twitches at 10 and 100 Hz, the ISR configuration only lead to a reduction of the twitch at 10 Hz. Thus, TS induced a proportionately greater loss of force at low stimulation frequency (10 Hz) compared with high stimulation frequency (100 Hz). Low frequency fatigue results from increases in intracellular free Ca^2+^ ([Ca^2+^i]) during fatigue and these elevations in [Ca^2+^i] activate processes which lead to a failure of excitation-contraction (E-C) coupling and Ca^2+^ release [35]. It is possible that TS induced a high failure of Ca^2+^ release that could affect the shape of the force-Ca^2+^ curve for both low and high frequencies. In contrast, ISR configuration may affect the steep part of the force-Ca^2+^ curve, where moderate falls in the Ca^2+^ release produce a greater loss of tension at low frequencies [36]. In summary, using peripheral measurements, the current study demonstrates that ISR is associated with less impairment in the muscle contraction properties compared with the TS configuration.

Our voluntary activation findings demonstrate that TS, but not ISR, induced an impairment in the voluntary neural drive to the knee extensors, even though the work-to-rest ratios were equated for both configurations. In addition, the TS configuration induced a significant reduction in the MEP amplitude (normalized to M-wave), suggesting a lower corticospinal excitability after the TS compared with ISR configuration. Moreover, the SICI reduction suggests that mechanisms acting at the cortical level may contribute to the central fatigue that is induced by the TS configuration. These mechanisms are likely to involve cortical GABA_A_ circuits, since SICI is related to activity in intracortical inhibitory circuits that use GABA_A_ as neurotransmitters [21] and is mediated at cortical rather than subcortical structures [21]. The observed reduction of the SICI may reflect compensatory mechanisms in response to the impairment in the central neural drive [14,17]. In order to maintain the target force, the brain plasticity induces an expansion of the motor areas by reducing the interneurons inhibitory activity, thus increasing the neural drive to the muscles [14].

The voluntary activation for the ISR configuration decreased from 90% to 84%. Although, this decrease was not significant, it is possible that central fatigue played a minor role in the MVC decrease, as indicated by the reduction of the MEP amplitude. In addition, the absence of changes in the SICI after the ISR configuration could be due to a fast recovery of the SICI circuit as a result of a less fatiguing protocol. This is supported by other findings showing that recovery times of SICI values between 5–10 minutes are associated with fatiguing isometric contractions [14].

The ICF values after both set configurations remained unaffected. Although, the effect of exhaustive exercise on ICF is not clear, our results support previous findings suggesting that muscle fatigue does not affect cortical glutamatergic circuits [14].

Finally, although, MEP changes should be interpreted with caution [17], both our MEP and voluntary activation findings demonstrate that the distribution between effort and rest during a training session has a different impact on the central nervous system, with ISR having a lower impact on the central muscle fatigue mechanisms compared with TS.

### Muscle Fatigue and Cardiovascular Response

During the performance of both set configurations, the mean and maximal values of the blood pressure parameters (MAP, SBP and DBP) increased significantly in comparison with pre-exercise values. These parameters returned to baseline values 10 minutes after the exercise. The maximal values of these parameters during the exercise were significantly lower for the ISR compared with the TS configuration. In addition, the ISR configuration induced lower HR_mean_, HR_max_ and RPP values in comparison with the TS. These findings are in agreement with a previous study using dynamic contractions [8] and confirm that the intra-set rest configuration induces lower cardiovascular responses compared with a traditional set configuration.

This increase in the HR in the traditional set-configuration is likely the result of a parasympathetic withdrawal, as suggested by the strong high frequency (HF) power density decrease after exercise, while the low frequency band (LF) demonstrated a more moderate power density decrease [37]. These changes were accompanied by an impairment of the baroreflex mechanism during the TR configuration, suggesting higher cardiovascular stress for the TR in comparison with the ISR configuration. Reductions in the HF and LF together with a baroreflex impairment has been associated with high intensity resistance training [38]. However, since the intensity for both set configurations was identical (50% of MVC) other factors related with the distribution of the effort may explain the observed differential RPP modulation.

The relative periods of muscle contraction may explain not only the differences in neuromuscular fatigue but also the differential cardiovascular responses between the configurations. The increases in RPP and MAP in response to the TS configuration were associated with a decrease in the voluntary activation. In addition, after the performance of the TS configuration a decrease of the HF twitch correlated with a decrease of the LF. This neuromuscular and cardiovascular modulation induced by the traditional set may be mediated by central and peripheral mechanisms. This is supported by higher perceived effort that was reported by the subjects during the traditional set compared with ISR. These findings may be related to the central command that is sent to the muscles in order to maintain the force during the relative longer muscle contraction periods. A direct relationship between the perception of effort and the central command sent to the muscles has been previously reported [39]. Moreover, it has been suggested that the increase in blood pressure and heart rate during time to task failure of a low force contraction is due to the contribution of the central command [1]. Our results of the intracortical inhibition as measured by pair pulse TMS (SICI) support the role of the central command for the cardiovascular regulation. The reduction in the SICI after the TS configuration correlated with a decrease in the sensitivity of the baroreflex response. To the best of our knowledge, only one other study found a significant correlation between SICI and BRS [40]. The authors postulated that the unloading of the baroreceptor enhanced the cortical excitability due to increased noradrenergic, dopaminergic, and serotonergic function within the motor cortex, leading to a decrease in SICI mechanisms.

Peripheral mechanisms may also have an impact on the perceived effort. This is supported by our finding that peripheral fatigue (LFF and DB10Hz) is associated with a higher local perceived effort (RPE-leg). We suggest that longer muscle contraction periods may induce prolonged periods of restricted blood flow and of muscular ischemia. Muscle ischemia activates the exercise pressor reflex response via mechanical and metabolic stimulation (i.e. III/IV afferents), leading to excitation-coupling failure in muscle fibers, affecting their contractibility [11,12,17]. In addition, the III/IV muscle afferents may limit the circuits that generate motor cortical output [41] causing a suboptimal firing of the motoneurons. The afferent signals from the musculature reach superior centers such as the Nucleus Tractus Solitarii (NTS), which plays a role as an integration center in the cardiovascular response receiving inputs from musculature, baroreceptors and the central command, modulating the vagal tone and the subsequent cardiovascular response [42].

Importantly, the above described correlations were not observed for the ISR configuration. This may be due to the fact that ISR did not induce sufficient fatigue levels in order to induce a modulation in the central command, unlike the TS configuration.

To the best of our knowledge this is the first study that found correlations between neuromuscular and cardiovascular modulations in response to resistance exercise training, further studies are needed in order to understand the nature of these relationships.

### Limitations

Despite the strong correlations reported in this study between voluntary activation and cardiovascular responses, our results are limited to an isometric contraction regime. There is evidence that isometric contractions induce higher blood pressure compared with dynamic contractions [43]. Thus, it is of importance to ascertain whether the correlations observed in the current study generalize to non-isometric contractions. Previous studies using ISR and TS configurations during dynamic contractions [8] have reported modulations in the cardiovascular responses similar to those observed in the current study and thus, it is likely that the association between voluntary activation and cardiovascular responses is not limited to isometric contractions. Moreover, it is possible that isometric training using higher intensities (greater than 50% of MVC used in the current study) may induce stronger cardiovascular responses, specifically in the ISR configuration.

Another limitation is related to the TMS measures that were utilized. The TMS measures were not obtained immediately after completion of the exercise, and it is known that MEP and SICI recover quickly after exercise [14]. However, after the TS configuration, both of these parameters (obtained 7 minutes post-exercise) remained significantly low, suggesting a bigger impact on the central muscle fatigue mechanisms compared with the ISR configuration.

### Significance

The current study demonstrates that the set configuration has a key role in the modulation of the cardiovascular response and fatigue effects in resistance training. An ISR configuration has an advantage over TS since subjects are able to complete the same amount of work in the same time but with lower cardiovascular stress, lower neuromuscular impairment and a lower perceived effort compared with a traditional set configuration. This finding is of importance for clinicians and trainers, who would like to avoid an unnecessary elevation of blood pressure and heart rate in healthy adults during the performance of resistance exercise. A recent review pointed out the relevance of the use of isometric resistance training as a treatment for cardiovascular diseases [43]. In addition, a recent study has reported similar increments of thigh circumference after both TR and ISR configurations training [44]. Together with our current findings we suggest that training protocols, such as ISR, result in a reduction of the cardiovascular impact of the exercise, while preserving muscle growth. This could be of relevance for elderly people that suffer from chronic conditions associated with old age such as muscle sarcopenia and cardiovascular disease (i.e. hypertension, heart failure). However, more studies must be conducted to further explore differences in muscle and cardiovascular adaptations between TR and ISR configuration training in elderly and young subjects.

### Conclusion

Our study supports the hypothesis that there is a relationship between central fatigue and hemodynamic responses, such that the greater the central fatigue, the larger the hemodynamic response. Our findings show that this relationship is modulated by the set configuration. An intra-set rest configuration is associated with lower central and peripheral fatigue with subsequent lower loss in maximal force values as well as lower cardiovascular stress in comparison with a traditional set configuration with equal work, rest and work-rest ratio. The greater hemodynamic response in traditional set configuration seems to be related to the magnitude of the exercise pressor reflex response, the baroreflex modulation and the central command. These changes are associated with a lower voluntary activation, suggesting a relationship between central fatigue and cardiac stress. To our knowledge this is the first study that compared the central and peripheral fatigue, in combination with hemodynamic and cardiovascular measures, of two resistance exercise set configurations with equal work to rest ratios. The findings provide further insight into the physiological mechanisms underlying set configuration and its relevance in managing the effects of resistance exercise.

## Supporting Information

S1 Dataset Raw data of the manuscript. (XLSX)

## Abbreviations

½RRT: half relaxation time
ANOVA: analysis of variance
BP: blood pressure
BRS: baroreflex sensitvity
DBP: diastolic blood pressure
EMG: electromyography
HF: high frequency
HR: heart rate
ICF: intracortical facilitation
ISR: intra set rest
LF: low frequency
LFF: low frequency fatigue
MAP: mean arterial pressure
MVC: maximal voluntary force
MEP: motor evoked potential
RF: rectus femoris
RFD: rate of force development
RMT: resting motor threshold
RPP: rate pressure product
RRI: R-R interva
SBP: systolic blood pressure
SICI: short intracortical inhibition
ST: single twitch
TMS: transcranial magnetic stimulation
TS: traditional set
TTF: time to failure
VA: voluntary activation
